# Diffuse auroral precipitation driven by lower-band chorus second harmonics

**DOI:** 10.1038/s41467-023-36095-x

**Published:** 2023-01-27

**Authors:** Xiongdong Yu, Zhigang Yuan, Jiang Yu, Dedong Wang, Dan Deng, H. O. Funsten

**Affiliations:** 1 School of Electronic Information, Wuhan University, Wuhan, Hubei China; 2 Planetary Environmental and Astrobiological Research Laboratory (PEARL), School of Atmospheric Sciences, Sun Yat-sen University, Zhuhai, China; 3 GFZ German Research Center for Geosciences, Potsdam, Germany; 4 ISR Division, Los Alamos National Laboratory, Los, Alamos, NM USA

**Keywords:** Aurora, Magnetospheric physics

## Abstract

Diffuse aurora at the Earth’s high latitude regions is mainly caused by the low-energy (0.1–30 keV) electron precipitation which carries the major energy flux into the nightside upper atmosphere. Previous studies have demonstrated that combined scattering by the upper- and lower- band chorus waves acts as the dominant cause of diffuse auroral precipitation, but that is not necessarily the case as these two types of waves do not always occur simultaneously, with the lower-band more often. Here we report that the lower-band chorus satisfying the preferred condition can generate their second harmonics so as to trigger the diffuse auroral electron precipitation. We find that the lower-band chorus alone can only cause the precipitation of electrons greater than 4 keV, while the self-consistently generated second harmonic is weak but still able to result in the electron precipitation below 4 keV. The combined effect of those modes results in the observed pancake electron distributions and the diffuse aurora. Our results clearly demonstrate an alternative but universal mechanism of chorus-driven diffuse aurora in the Earth, which may also apply to the auroral formation in other planetary magnetospheres.

## Abstract (web summary)

Scattering by the upper- and lower-band chorus waves are the dominant cause of diffuse auroral precipitation. Here, the authors show that the lower-band chorus alone satisfies the preferred condition for the generation of second harmonics to trigger the diffuse auroral electron precipitation.

## Introduction

Low-energy electron precipitation has been demonstrated to be the primary cause of the diffuse aurora^1^. To precipitate into the upper atmosphere to collide with neutral atoms and molecules and then form aurora^2^, electrons need to be located inside the loss cone which is centered along the magnetic field and of a relatively small angle near the magnetospheric equator. As injected from the magnetotail plasma sheet into the inner magnetosphere^3^, most electrons are outside the loss cone and trapped between their mirror points, until they are subjected to scattering by waves that can violate the first adiabatic invariant and scatter particles into the loss cone^4^. One effective way for electrons to be scattered into the loss cone is to interact with the whistler-mode chorus waves through cyclotron resonances^5–7^. Combined scattering by the upper- and lower- band chorus, which are separated by 0.5*F*_ce_ (where *F*_ce_ is the electron gyrofrequency) in frequency range, has been demonstrated as the main cause for diffuse auroral electron precipitation^8–10^. However, lower-band chorus (LBC) waves often occur alone, not accompanied with upper-band chorus^11^, even when diffuse auroral electron precipitation as well as the remnant pancake distribution^12^ in space have been found. Spatial separation of upper- and lower- band chorus weakens the efficacy of such a mechanism, because electrons should take time to move across the spatial gap between. Therefore, we seek to find other potential alternatives to form diffuse aurora.

In this work, we show that lower-band chorus alone can trigger diffuse auroral precipitation through exciting their second harmonics when the preferred condition is satisfied.

## Results

### Observations

**Fig. 1:**
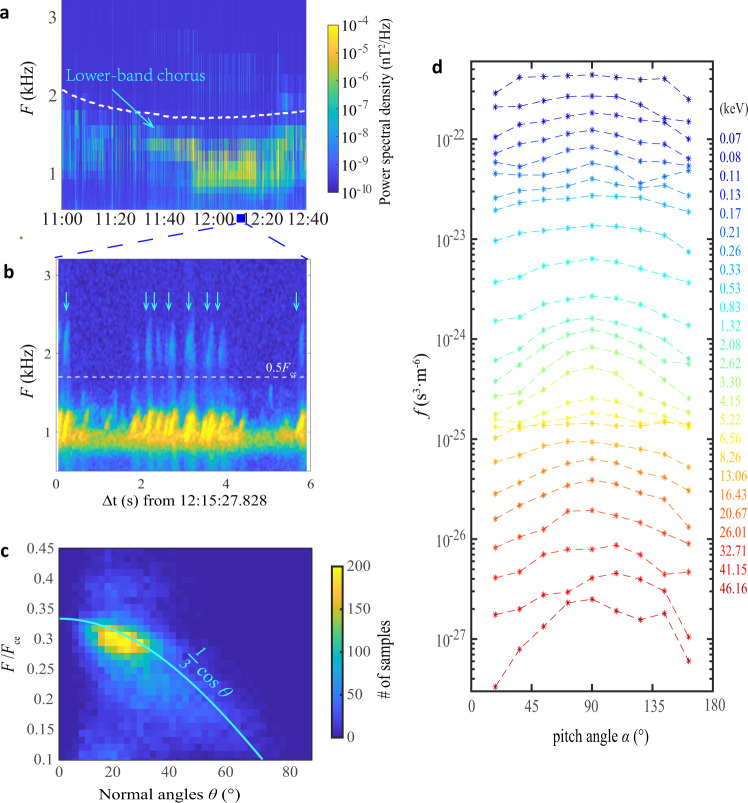
Van Allen Probe observations of diffuse auroral precipitation event on 14 January 2013. **a**, **b** chorus wave power spectral densities in the survey and burst modes measured by the Van Allen Probe A. The dashed white lines in **a** and **b** denote one-half electron gyrofrequencies (*F*_ce_), while light blue arrows in **b** indicate the second-harmonic emissions self-consistently generated by lower-band chorus waves. **c** Statistical survey of the wave frequency and normal angles of the lower-band chorus waves in second-harmonic events, which is shown to fall around the preferred condition marked by the light blue curve. **d** Electron space distributions during the time interval shown in **b**. Pancake distributions, identified as the peaked fluxes around a pitch angle of 90°, are captured, especially in energies during 0.5–4 keV, which is verified to be the result of those observed second-harmonic emissions exhibited in **b**.

**Fig. 2:**
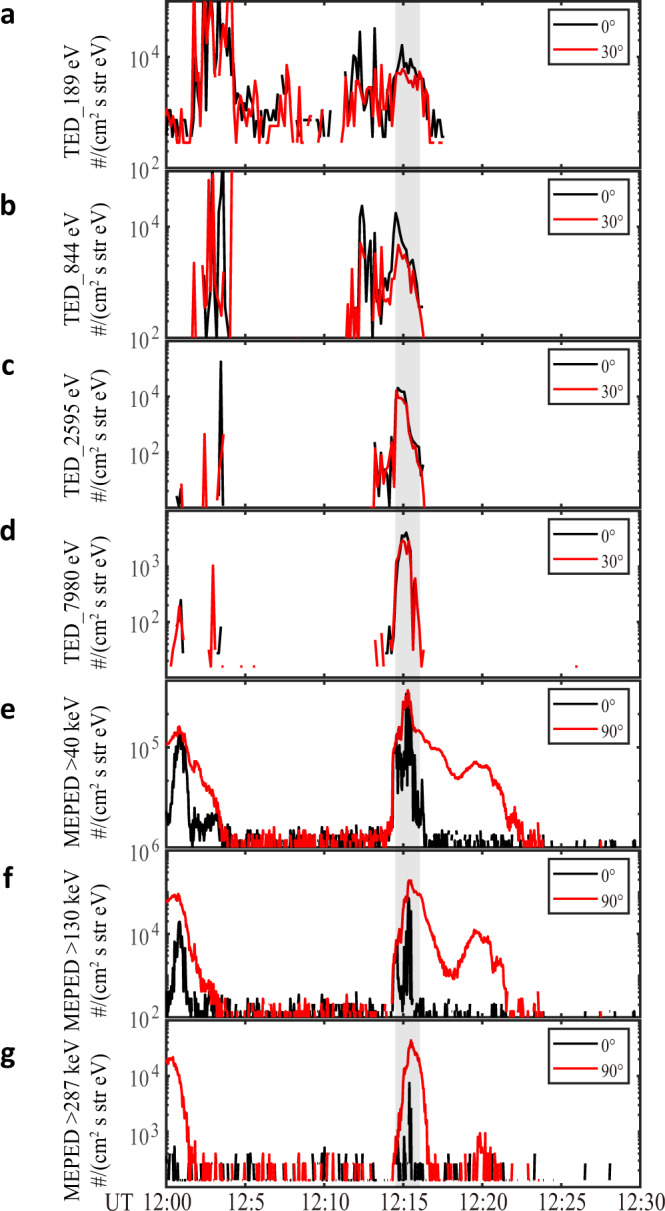
NOAA 18 observations of diffuse auroral precipitation event on 14 January 2013. **a**–**d** Precipitating electron fluxes in different energy channels (189 eV, 844 eV, 2.595 keV, and 7.980 keV, respectively) measured by the Total Energy Detector (TED) onboard the National Oceanic and Atmospheric Administration Polar Orbiting Environmental Satellite 18 (NOAA 18) in the ionospheric position conjugated with the Van Allen Probe A. The black and red curves in **a**–**d** denote the data measured by the 0° and 30° detectors, respectively. **e**–**g** Precipitating electron fluxes in different energy channels (>40 keV, >130 keV, and >287 keV, respectively) measured by the Medium Energy Proton and Electron Detector (MEPED) onboard NOAA 18. The black and red curves in **e**–**g** denote the data measured by the 0° and 90° detectors, respectively. The gray rectangle indicates the electron diffuse auroral precipitation observed by NOAA 18 in the ionospheric position conjugated with Van Allen Probe A, which has detected chorus waves and their second-harmonic near the magnetospheric equator.

Figure 1a shows such a typical case where lower-band chorus waves are captured with weak second-harmonic (SH) emissions (light blue arrows in Fig. 1b), instead of traditional upper-band chorus waves, by the Van Allen Probe A^13^ during 1100−1240 UT on January 14, 2013. Data samples of tri-axis magnetic fields (B_u_, B_v_, B_w_)^14^ and electric fields in the spin plane (E_u_, E_v_)^15^ measured in a burst-mode are illustrated in Supplementary Fig. 1a. The observed electric field components (peak-to-peak) are only about 1/40 of the range of EFW instrument, but as shown in Supplementary Fig. 1b, weak SHs are found to occur in a same way as in the magnetic field one (cf. Figure 1b), suggesting that reasonable nonlinear wave processes in plasmas are involved in these data samples. Note that though the power spectral densities of SH (~10^−8^ nT^2^/Hz, Supplementary Fig. 1c) are much lower than the fundamental, but still higher than the noise level by two orders of magnitude (below 10^−10^ nT^2^/Hz). These SH emissions are excited by lower-band chorus waves through coherent nonlinear processes^16,17^, and then they usually own a double frequency and wave vector as those of lower-band chorus waves, from which we can identify these emissions via bicoherence index (Supplementary Fig. 2). Since the SH are often weak, efforts have been made to confirm that they are natural emissions resulting from nonlinear physical process (see Methods, Data samples, and instrumental effect exclusion). Even if the traditional upper-band chorus is absent, the electron pancake distribution is still observed (Fig. 1d), especially near 2−3 keV, which is the remnant portion left in space as those inside loss cone have crossed the ionosphere and move into the upper atmosphere, resulting in the diffuse aurora. These precipitating electrons have been observed by National Oceanic and Atmospheric Administration (NOAA) satellite 18 in an altitude of ~880 km (Fig. 2), which together with the Van Allen Probe A provide a good conjugated observation (Supplementary Fig. 3). It suggests the potential role of SH emissions playing in the diffuse auroral precipitation. Note that the case shown in Fig. 1 is not an accidently observational event, and another similar event has been shown in Supplementary Fig. 4 and Supplementary Fig. 5. In this case, NOAA 19 (denoted as the black curve in Supplementary Fig. 4a) has observed electron precipitations near 03:58 (marked by the gray rectangle in Supplementary Fig. 5). Simultaneously, Van Allen Probe A (marked by the red rectangle in Supplementary Fig. 4a) has detected lower-band chorus waves and their SH between 03:40 UT and 04:10 UT (see Supplementary Fig. 4b, c), suggesting that magnetospheric electrons can be scattered into loss cones by chorus waves and their SHs so that they can precipitate into the atmosphere.

To determine whether SH emissions contribute to diffuse aurora, we should estimate the scattering effect of SH emissions on electrons. Generally, when viewing multiple periods of wave fields in their trajectories, electrons will be forced by whistler-mode waves into stochastic motions in phase space, leading to resonant diffusions in pitch angles and momentum^18^. The effects of whistler-mode waves on the evolution of the electron distribution are often estimated by calculating the resonant diffusion rates^19–21^. Previous studies have demonstrated that SHs of chorus are a very common phenomenon and widely observed in the terrestrial magnetosphere^22^, especially in the regions where the most intense diffuse auroral precipitation is found. After statistically analyzing all high-resolution data from the Van Allen Probe A, we find that SHs are easily generated when the preferred condition (*F*/*F*_ce_ = cos*θ*/3, see Methods, Preferred condition for SH generation of lower-band chorus, for detailed derivations) for lower-band chorus waves is satisfied (light blue curve in Fig. 1c), which means that SHs are located in or near the inherent modes. This allows us to roughly treat SH emissions also as whistler-mode waves when estimating their scattering on electrons.

### Fokker–Planck simulations

**Fig. 3:**
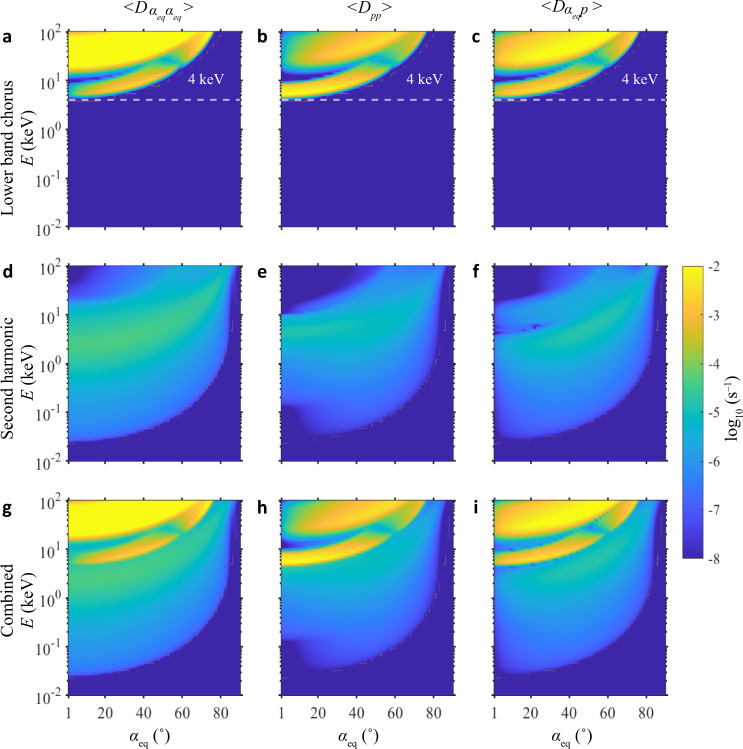
Bounce-averaged pitch-angle (<${D}_{{{\rm{\alpha }}}_{{eq}}{\alpha }_{{eq}}}$>), momentum (<*D*_pp_>), and mixed (<${D}_{{{\rm{\alpha }}}_{{eq}}p}$>) diffusion rates as a function of electron energy (E) and equatorial pitch angle (*α*_eq_). **a**–**c** Lower-band chorus only cause electron diffusions in energy above 4 keV (dashed white lines). **d**–**f** Second-harmonic emissions can only cause effective diffusion at a wide range of energy centered near 2 keV. **g**–**i** Combined effect of lower-band chorus and their second harmonic can cause electron diffusions at energy during 0.1–30 keV, which contributes to diffuse auroral precipitations.

Figure 3 shows the bounce-averaged electron pitch-angle (left column), momentum (middle column), mixed (right column) diffusion rates for lower-band chorus only (top), SHs only (middle), and combined lower-band chorus and their SHs (bottom) under the plasma environment and wave parameters of the event displayed in Fig. 1. It is clearly shown that lower-band chorus can cause rapid diffusion of electrons only above 4 keV (Fig. 3a–c), but SHs is effective for electron scattering over a wide range of pitch angles including the loss cone at a wide range of energy centered near 2 keV (Fig. 3d–f). It is the weak SH that is suggested to form the observed electron pancake distribution near 2−3 keV (Fig. 1d). Then combined effect of lower-band chorus and SHs contributes to the total diffuse auroral precipitation of electrons during 0.1−30 keV (Fig. 3g–i).

**Fig. 4:**
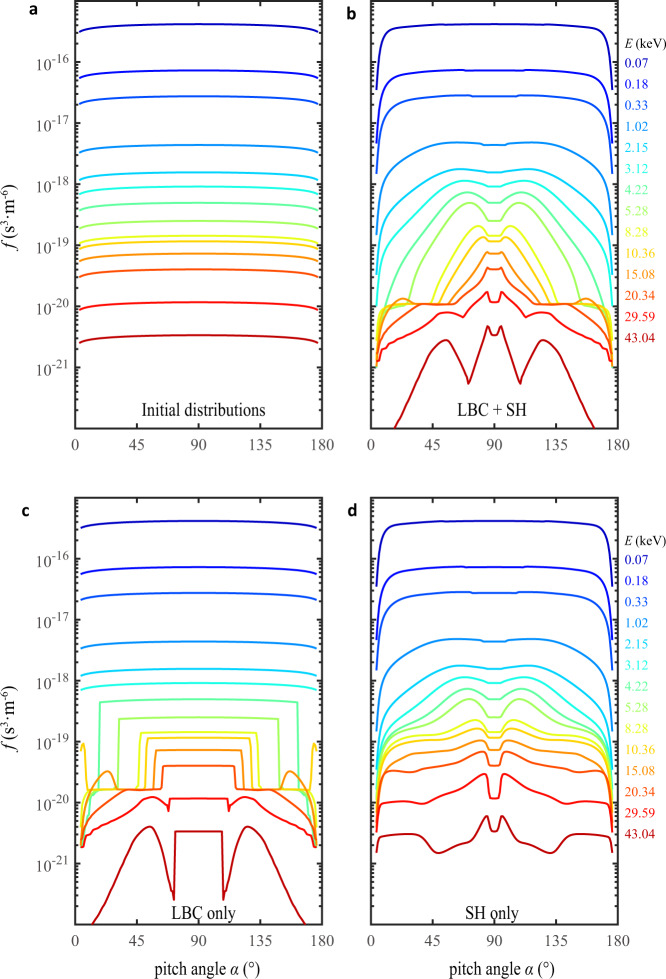
Evolution of electron phase space distributions. **a** Initially electron phase space distribution (*f*) is assumed to be rather isotropic, which is correspondingly deformed under 2-hour scattering effect of different waves. **b** Combined diffusion of LBC and SH eventually forms the pancake distribution, after electrons near the loss cone are rapidly driven into the ionosphere. **c** Lower-band chorus (LBC) only cause rapid loss of electrons above 4 keV with trapped electrons affected slightly. **d** Second-harmonic (SH) emissions alone contribute to the evolutions of almost all stimulated electrons, especially near 2 keV.

To study the total effect of lower-band chorus and SHs on forming electron pancake distributions, we solve the Fokker–Planck equation^23–25^ with the diffusion rates shown in Fig. 3. The initial electron distribution is assumed to be rather isotropic (Fig. 4a), which turns into a pancake distribution in the timescale of hours under the combined diffusion of lower-band chorus and their SHs (Fig. 4b). Although lower-band chorus act predominately on electrons above 4 keV (Fig. 4c), the SH emissions can resonant with the entire simulated populations and dominate the effect on electrons below 4 keV (Fig. 4d).

## Discussion

Lower-band chorus waves are often observed alone and are not accompanied by the upper-band chorus waves, which may be due to the fact that they are more easily excited by the anisotropic electrons injected from the plasma sheet. When propagating along their trajectories in the magnetosphere^26^, those lower-band chorus waves would pass the regions where the preferred condition is satisfied to generate their SHs. Subsequently, the generated SHs, playing the role of traditional upper-band chorus demonstrated in previous models, can work together with lower-band chorus waves to drive diffuse auroral electron precipitations.

**Fig. 5:**
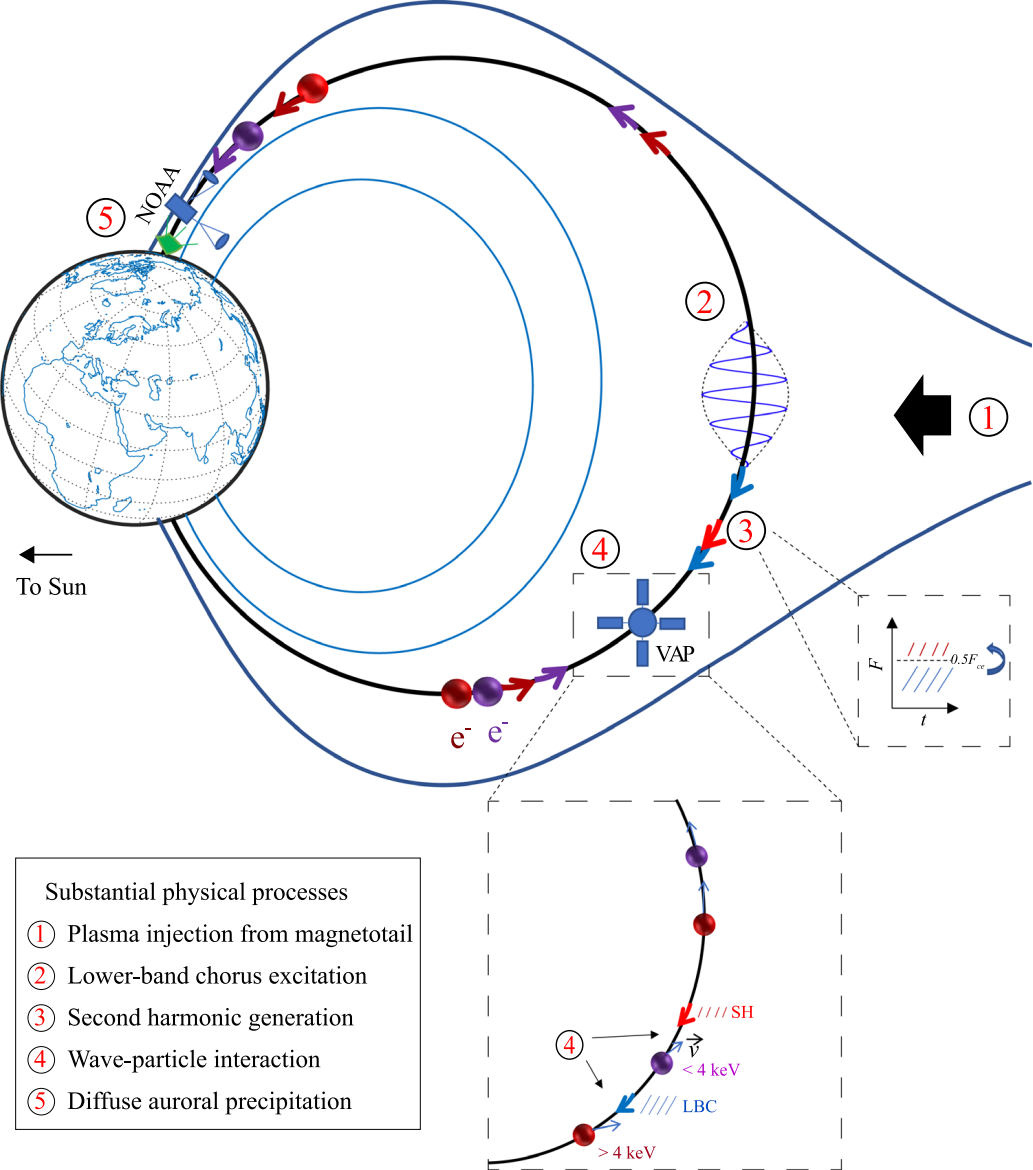
Schematic diagram of electron precipitation driven by LBC and SH. After injected from magnetotail (step 1), fresh electrons become anisotropic to excite lower-band chorus (LBC, step 2). Lower-band chorus can self-consistently generate their second harmonic (SH) emissions (step 3, observed by the Van Allen Probe mission, VAP), and then they work together (step 4) to cause electron precipitations both above and below 4 keV to form diffuse aurora (step 5, observed by the National Oceanic and Atmospheric Administration Polar Orbiting Environmental Satellite, NOAA). Balls with bright red colors denote electrons with energies >4 keV while those with dark red colors are below 4 keV.

We have shown that the combined diffusion of lower-band chorus and their SHs can be an alternate candidate causing diffuse auroral precipitations. As illustrated in Fig. 5, lower-band chorus, excited by the injected electrons from the central plasma sheet, self-consistently generates their SHs and then scatter electrons in a combined way. As a result, the scattered electrons may move to ionosphere and lead to ionization of neutral atoms and molecules in the upper atmosphere, causing diffuse aurora. The physical process presented here may be relevant to the physics of the formation of diffuse aurora in Jupiter’s^27^, Saturn’s^28^ magnetosphere, and other planetary magnetospheres.

## Methods

### Data samples and instrumental effect exclusion

To exclude the instrumental effect, we have checked the burst-mode data samples of magnetic fields and the electric fields during the time interval shown in Fig. 1b (see Supplementary Fig. 1). Another evidence supporting the fact that the SH results from natural nonlinear wave processes in space plasmas is also shown in Supplementary Fig. 6. In this case (observed by the Van Allen Probe A near 00:16:31 UT on February 24, 2014), several large-amplitude chorus waves are observed, but the SH phenomenon only occurs for the element near 00:16:36 UT (marked by the gray rectangles in panels a and b). It is clearly shown in panel (a) that the element accompanied with SH owns an amplitude (~0.15 nT) slightly smaller than that in the beginning (~0.20 nT), suggesting that the amplitude of the fundamental is not the only factor controlling the generation of these SHs. In other words, a fundamental wave with a large amplitude is not necessarily easier to excite the SH than that with a small amplitude. Note that if the SH is due to instrumental effects, those chorus elements with a large amplitude should be accompanied with SHs, not the other way around. As shown in the bottom of panel (b), the chorus element exciting the SH owns a power-weighted normal angle of ~43°, which is larger than those of other elements (<~20°). Note that the frequency of the fundamental wave is ~1 kHz, and the electron gyrofrequency is about 4.486 kHz. According to the preferred condition for the SH generation (*F*/*F*_*ce*_ = cos*θ*/3), the preferred normal angle for the chorus to excite SHs is ~48°. Consequently, the one element with a normal angle of ~43° will be easier to excite SHs than those with normal angles of <~20°, which is consistent with the satellite observations. That is to say, the generation of the observed SHs satisfies the theoretic prediction that results from nonlinear wave processes^17,29^. It is reasonable to demonstrate that the observed SHs are due to nonlinear wave processes in space plasmas, rather than instrumental effects.

Moreover, we have estimated the theoretical values of the amplitude ratio of the SH to the fundamental wave for the case shown in Fig. 1b (see Supplementary Fig. 7) under a cold plasma approximation^17,29^, using the observed plasma parameters as well as the amplitude of the fundamental wave. The calculated amplitude ratios of the second to fundamental harmonics (*B*_2_/*B*_1_) are displayed as a function of the normalized wave frequency (*F*/*F*_*ce*_) and wave normal angles (*θ*) in Supplementary Fig. 7a. It is clearly shown that the ratio becomes significantly large only if the wave frequency and normal angle satisfy the preferred condition (*F*/*F*_*ce*_ = cos*θ*/3). Panel (b) exhibits the profile at *θ* = 25°, which owns a maximum at *F*/*F*_*ce*_ = 0.3, in consistent with the observed fundamental chorus waves. The maximum *B*_2_/*B*_1_ shown in panel (b) is about 1.04% while the observed one is about 1.46%, that is, the observed ratio is just a bit larger than the theoretical value. The observed SH is roughly consistent with the theoretical prediction, suggesting that they are excited by the fundamental through nonlinear wave processes. Consequently, we believe that these SHs are natural emissions driven by the lower-band chorus waves through nonlinear wave processes^17^.

### Bicoherence index

Bicoherence index is often used to diagnose the phase relation during wave-wave couplings^22^, which is given by ${\left|\left\langle {\widetilde{{{{{{\boldsymbol{E}}}}}}}}_{{{{{{\boldsymbol{z}}}}}}}\left({{{{{{\boldsymbol{F}}}}}}}_{{{{{{\boldsymbol{a}}}}}}}\right){\widetilde{{{{{{\boldsymbol{B}}}}}}}}_{{{{{{\boldsymbol{y}}}}}}}\left({{{{{{\boldsymbol{F}}}}}}}_{{{{{{\boldsymbol{b}}}}}}}\right){\widetilde{{{{{{\boldsymbol{B}}}}}}}}_{{{{{{\boldsymbol{y}}}}}}}^{*}\left({{{{{{\boldsymbol{F}}}}}}}_{{{{{{\boldsymbol{c}}}}}}}\right)\right\rangle \right|}^{{{{{{\bf{2}}}}}}}/{\left|\left\langle {\widetilde{{{{{{\boldsymbol{E}}}}}}}}_{{{{{{\boldsymbol{z}}}}}}}\left({{{{{{\boldsymbol{F}}}}}}}_{{{{{{\boldsymbol{a}}}}}}}\right){\widetilde{{{{{{\boldsymbol{B}}}}}}}}_{{{{{{\boldsymbol{y}}}}}}}\left({{{{{{\boldsymbol{F}}}}}}}_{{{{{{\boldsymbol{b}}}}}}}\right)\right\rangle \right|}^{{{{{{\bf{2}}}}}}}{\left|\left\langle {\widetilde{{{{{{\boldsymbol{B}}}}}}}}_{{{{{{\boldsymbol{y}}}}}}}^{*}\left({{{{{{\boldsymbol{F}}}}}}}_{{{{{{\boldsymbol{c}}}}}}}\right)\right\rangle \right|}^{{{{{{\bf{2}}}}}}}$ with ${{{{{{\boldsymbol{F}}}}}}}_{{{{{{\boldsymbol{c}}}}}}}={{{{{{\boldsymbol{F}}}}}}}_{{{{{{\boldsymbol{a}}}}}}}+{{{{{{\boldsymbol{F}}}}}}}_{{{{{{\boldsymbol{b}}}}}}}$. Here $\widetilde{{{{{{\boldsymbol{E}}}}}}}$ and $\widetilde{{{{{{\boldsymbol{B}}}}}}}$ denote the electric and magnetic spectra, whose frequencies are ${{{{{{\boldsymbol{F}}}}}}}_{{{{{{\boldsymbol{a}}}}}}}$ and ${{{{{{\boldsymbol{F}}}}}}}_{{{{{{\boldsymbol{b}}}}}}}$, respectively. The asterisk represents a conjugation operator, and the bracket < > denotes an average during the time interval of interest. For the bicoherence index, an absolute value near one indicates strong couplings, while a near-zero value suggests independent wave behaviors. To obtain the bicoherence index, the detected burst-mode magnetic and electric field data for the event shown in Fig. 1 are used. Due to the necessary condition for the SH generation^17^, high bicoherence index should be found near the frequency of lower-band chorus (i.e., 1000 Hz in our event) in both abscissa and ordinate, as shown in Supplementary Fig. 2. This also indicates these SHs are resulted from nonlinear processes.

### Conjugated observation of Van Allen Probe A and NOAA 18

As entering the loss cone near the magnetospheric equator, electrons would move inward high-latitude regions along the magnetic field line and then be detected by satellites in the ionosphere. To capture such a process, satellites in the magnetosphere and ionosphere should be located in the same magnetic flux tube and conjugate with each other. Mapping along with magnetic field lines has previously been done in a number of studies^5,7,30–32^ to confirm such a conjugate configuration for satellites. Here using TS04D magnetic field model^33^, we have mapped the two satellites (Van Allen Probe A and NOAA 18) along the magnetic field line into a region at an altitude of 100 km (Supplementary Fig. 3) to show their conjugate configuration. We have also checked footprints of Van Allen Probe A with T89^34^ magnetic field models and found only slight difference in the latitude and longitude of footprints (a maximum latitudinal difference of 0.1° and a maximum longitudinal difference of 0.5°), as illustrated in Supplementary Fig. 8. It indicates that in the event shown in Fig. 1, Van Allen Probe A and NOAA 18 have footprints close to each other, forming a conjugated observation.

### Preferred condition for SH generation of lower-band chorus

Previous studies^17^ have revealed that wave equations describing the fundamental and SHs can be given by

1 $${{{{{\bf{D}}}}}}\left({{{{{{\boldsymbol{\omega }}}}}}}_{{{{{{\bf{1}}}}}}},{{{{{{\bf{k}}}}}}}_{{{{{{\bf{1}}}}}}}\right) \cdot {{{{{{\bf{E}}}}}}}_{{{{{{\bf{1}}}}}}}\left({{{{{{\boldsymbol{\omega }}}}}}}_{{{{{{\bf{1}}}}}}},{{{{{{\bf{k}}}}}}}_{{{{{{\bf{1}}}}}}}\right)=\left[{{{{{\boldsymbol{\varepsilon }}}}}}\left({{{{{{\boldsymbol{\omega }}}}}}}_{{{{{{\bf{1}}}}}}},{{{{{{\bf{k}}}}}}}_{{{{{{\bf{1}}}}}}}\right)-\frac{{{{{{{\boldsymbol{c}}}}}}}^{{{{{{\bf{2}}}}}}}{{{{{{\boldsymbol{k}}}}}}}_{{{{{{\bf{1}}}}}}}^{{{{{{\bf{2}}}}}}}}{{{{{{{\boldsymbol{\omega }}}}}}}_{{{{{{\bf{1}}}}}}}^{{{{{{\bf{2}}}}}}}}{{{{{\boldsymbol{I}}}}}}+\frac{{{{{{{\boldsymbol{c}}}}}}}^{{{{{{\bf{2}}}}}}}}{{{{{{{\boldsymbol{\omega }}}}}}}_{{{{{{\bf{1}}}}}}}^{{{{{{\bf{2}}}}}}}}{{{{{{\bf{k}}}}}}}_{{{{{{\bf{1}}}}}}}{{{{{{\bf{k}}}}}}}_{{{{{{\bf{1}}}}}}}\right] \cdot {{{{{{\bf{E}}}}}}}_{{{{{{\bf{1}}}}}}}\left({{{{{{\boldsymbol{\omega }}}}}}}_{{{{{{\bf{1}}}}}}},{{{{{{\bf{k}}}}}}}_{{{{{{\bf{1}}}}}}}\right)={{{{{\bf{0}}}}}}$$

2 $${{{{{\bf{D}}}}}}\left({{{{{{\boldsymbol{\omega }}}}}}}_{{{{{{\bf{2}}}}}}},{{{{{{\bf{k}}}}}}}_{{{{{{\bf{2}}}}}}}\right) \cdot {{{{{{\bf{E}}}}}}}_{{{{{{\bf{2}}}}}}}\left({{{{{{\boldsymbol{\omega }}}}}}}_{{{{{{\bf{2}}}}}}},{{{{{{\bf{k}}}}}}}_{{{{{{\bf{2}}}}}}}\right) 	=\left[{{{{{\boldsymbol{\varepsilon }}}}}}\left({{{{{{\boldsymbol{\omega }}}}}}}_{{{{{{\bf{2}}}}}}},{{{{{{\boldsymbol{k}}}}}}}_{{{{{{\bf{2}}}}}}}\right)-\frac{{{{{{{\boldsymbol{c}}}}}}}^{{{{{{\bf{2}}}}}}}{{{{{{\boldsymbol{k}}}}}}}_{{{{{{\bf{2}}}}}}}^{{{{{{\bf{2}}}}}}}}{{{{{{{\boldsymbol{\omega }}}}}}}_{{{{{{\bf{2}}}}}}}^{{{{{{\bf{2}}}}}}}}{{{{{\boldsymbol{I}}}}}}+\frac{{{{{{{\boldsymbol{c}}}}}}}^{{{{{{\bf{2}}}}}}}}{{{{{{{\boldsymbol{\omega }}}}}}}_{{{{{{\bf{2}}}}}}}^{{{{{{\bf{2}}}}}}}}{{{{{{\bf{k}}}}}}}_{{{{{{\bf{2}}}}}}}{{{{{{\bf{k}}}}}}}_{{{{{{\bf{2}}}}}}}\right] \cdot {{{{{{\bf{E}}}}}}}_{{{{{{\bf{2}}}}}}}\left({{{{{{\boldsymbol{\omega }}}}}}}_{{{{{{\bf{2}}}}}}},{{{{{{\bf{k}}}}}}}_{{{{{{\bf{2}}}}}}}\right)\\ 	=-\frac{{{{{{\boldsymbol{i}}}}}}{{{{{\bf{4}}}}}}{{{{{\boldsymbol{\pi }}}}}}}{{{{{{{\boldsymbol{\omega }}}}}}}_{{{{{{\bf{1}}}}}}}}{{{{{{\bf{J}}}}}}}_{{{{{{\boldsymbol{NL}}}}}}}$$

where ${{{{{\boldsymbol{\omega }}}}}}$ and ${{{{{\bf{k}}}}}}$ are the wave angular frequency and vector, *c* and ${{{{{\boldsymbol{I}}}}}}$ are the speed of light and the identity matrix, and ${{{{{\bf{E}}}}}}$ is the electric field. Note that quantities with subscripts 1 are related to the fundamental wave, while those with subscripts 2 are for the SH. Additionally, ${{{{{\boldsymbol{\varepsilon }}}}}}\left({{{{{\boldsymbol{\omega }}}}}}{{{{{\boldsymbol{,}}}}}}{{{{{\bf{k}}}}}}\right)$ is the dielectric tensor, and ${{{{{\bf{D}}}}}}\left({{{{{\boldsymbol{\omega }}}}}}{{{{{\boldsymbol{,}}}}}}{{{{{\bf{k}}}}}}\right)$ is the coefficient matrix, both of them have a general expression^17,35^. ${{{{{{\bf{J}}}}}}}_{{{{{{\boldsymbol{NL}}}}}}}$ is the nonlinear current density resulting from the interactions between the fundamental wave and background plasmas, which acts as the driving source for the SH.

Following standard methods in nonlinear plasma theory^17,36^, one can obtain that

3 $${{{{{\mathbf{exp }}}}}}\left[{{{{{\boldsymbol{i}}}}}}\left({{{{{{\bf{k}}}}}}}_{{{{{{\bf{2}}}}}}} \cdot {{{{{{\bf{r}}}}}}}_{{{{{{\bf{g}}}}}}}-{{{{{{\boldsymbol{\omega }}}}}}}_{{{{{{\bf{2}}}}}}}{{{{{\boldsymbol{t}}}}}}\right)\right]{{{{{{\boldsymbol{A}}}}}}}_{{{{{{\bf{2}}}}}}}={{{{{\mathbf{exp }}}}}}\left[{{{{{\boldsymbol{i}}}}}}{{{{{\bf{2}}}}}}\left({{{{{{\bf{k}}}}}}}_{{{{{{\bf{1}}}}}}} \cdot {{{{{{\bf{r}}}}}}}_{{{{{{\bf{g}}}}}}}-{{{{{{\boldsymbol{\omega }}}}}}}_{{{{{{\bf{1}}}}}}}{{{{{\boldsymbol{t}}}}}}\right)\right]{{{{{{\boldsymbol{A}}}}}}}_{{{{{{\bf{1}}}}}}}$$

where ${{{{{{\bf{r}}}}}}}_{{{{{{\bf{g}}}}}}}$ is the guiding center variable, while ${{{{{{\boldsymbol{A}}}}}}}_{{{{{{\boldsymbol{1}}}}}}}$ and ${{{{{{\boldsymbol{A}}}}}}}_{{{{{{\boldsymbol{2}}}}}}}$ are factors concerning the first-order and second-order particle distribution functions, respectively. This equation has a nontrivial solution for

4 $${{{{{{\boldsymbol{\omega }}}}}}}_{{{{{{\bf{2}}}}}}}={{{{{\bf{2}}}}}}{{{{{{\boldsymbol{\omega }}}}}}}_{{{{{{\bf{1}}}}}}}\,{{{{{\bf{and}}}}}}\,{{{{{{\bf{k}}}}}}}_{{{{{{\bf{2}}}}}}}={{{{{\bf{2}}}}}}{{{{{{\bf{k}}}}}}}_{{{{{{\bf{1}}}}}}}$$

which is the necessary condition for the SH generation.

The electric field of the excited SH (${{{{{{\bf{E}}}}}}}_{{{{{{\boldsymbol{2}}}}}}}$) can be solved from Eq. (2)

5 $${{{{{{\bf{E}}}}}}}_{{{{{{\bf{2}}}}}}}=-\frac{{{{{{\boldsymbol{i}}}}}}{{{{{\bf{4}}}}}}{{{{{\boldsymbol{\pi }}}}}}}{{{{{{{\boldsymbol{\omega }}}}}}}_{{{{{{\bf{1}}}}}}}}{{{{{\bf{U}}}}}}{{{{{{\boldsymbol{\Lambda }}}}}}}^{-{{{{{\bf{1}}}}}}}{{{{{{\bf{U}}}}}}}^{{{{{{\boldsymbol{T}}}}}}}{{{{{{\bf{J}}}}}}}_{{{{{{\boldsymbol{NL}}}}}}}$$

where ${{{{{\bf{U}}}}}}$ and ${{{{{\boldsymbol{\Lambda }}}}}}$ are the matrices obtained from the spectral decomposition of the coefficient matrix in the left-hand side of Eq. (2) via ${{{{{\bf{D}}}}}}\left({{{{{{\boldsymbol{\omega }}}}}}}_{{{{{{\bf{2}}}}}}}{{{{{\boldsymbol{,}}}}}}{{{{{{\bf{k}}}}}}}_{{{{{{\bf{2}}}}}}}\right){{{{{\boldsymbol{=}}}}}}{{{{{{\bf{U}}}}}}}^{{{{{{\bf{T}}}}}}}{{{{{\boldsymbol{\Lambda }}}}}}{{{{{\bf{U}}}}}}$. Specifically, ${{{{{\bf{U}}}}}}$ consists of eigenvectors of ${{{{{\bf{D}}}}}}\left({{{{{{\boldsymbol{\omega }}}}}}}_{{{{{{\bf{2}}}}}}}{{{{{\boldsymbol{,}}}}}}{{{{{{\bf{k}}}}}}}_{{{{{{\bf{2}}}}}}}\right)$, and ${{{{{\boldsymbol{\Lambda }}}}}}$ is the diagonal matrix whose elements are the corresponding eigenvalues (say, ${{{{{{\boldsymbol{\lambda }}}}}}}_{{{{{{\boldsymbol{1}}}}}}}{{{{{\boldsymbol{,}}}}}}{{{{{{\boldsymbol{\lambda }}}}}}}_{{{{{{\boldsymbol{2}}}}}}}{{{{{\boldsymbol{,}}}}}}{{{{{\bf{and}}}}}}\,{{{{{{\boldsymbol{\lambda }}}}}}}_{{{{{{\boldsymbol{3}}}}}}}$). Then ${{{{{{\boldsymbol{\Lambda }}}}}}}^{{{{{{\boldsymbol{-}}}}}}{{{{{\bf{1}}}}}}}$ is also a diagonal matrix whose elements are ${{{{{{\boldsymbol{\lambda }}}}}}}_{{{{{{\boldsymbol{1}}}}}}}^{{{{{{\boldsymbol{-}}}}}}{{{{{\boldsymbol{1}}}}}}}{{{{{\boldsymbol{,}}}}}}\,{{{{{{\boldsymbol{\lambda }}}}}}}_{{{{{{\boldsymbol{2}}}}}}}^{{{{{{\boldsymbol{-}}}}}}{{{{{\boldsymbol{1}}}}}}}{{{{{\boldsymbol{,}}}}}}\,{{{{{\bf{and}}}}}}\,{{{{{{\boldsymbol{\lambda }}}}}}}_{{{{{{\boldsymbol{3}}}}}}}^{{{{{{\boldsymbol{-}}}}}}{{{{{\boldsymbol{1}}}}}}}$. Equation (5) demonstrates that the SH would own a large enough power (so that they can be captured from the noise level) when ${{{{{{\bf{J}}}}}}}_{{{{{{\boldsymbol{NL}}}}}}}$ is significantly large or there exists some eigenvalues nearly or even exactly equaling to zeros (so that ${{{{{{\boldsymbol{\lambda }}}}}}}_{{{{{{\boldsymbol{1}}}}}}}^{{{{{{\boldsymbol{-}}}}}}{{{{{\boldsymbol{1}}}}}}}{{{{{\boldsymbol{,}}}}}}{{{{{{\boldsymbol{\lambda }}}}}}}_{{{{{{\boldsymbol{2}}}}}}}^{{{{{{\boldsymbol{-}}}}}}{{{{{\boldsymbol{1}}}}}}}{{{{{\boldsymbol{,}}}}}}{{{{{\bf{or}}}}}}{{{{{{\boldsymbol{\lambda }}}}}}}_{{{{{{\boldsymbol{3}}}}}}}^{{{{{{\boldsymbol{-}}}}}}{{{{{\boldsymbol{1}}}}}}}$ would become significantly large)^17,29^. The latter means a vanishing determinant of ${{{{{\bf{D}}}}}}\left({{{{{{\boldsymbol{\omega }}}}}}}_{{{{{{\bf{2}}}}}}}{{{{{\boldsymbol{,}}}}}}{{{{{{\bf{k}}}}}}}_{{{{{{\bf{2}}}}}}}\right)$, that is, ${{{{{\mathbf{det }}}}}}\,{{{{{\bf{D}}}}}}\left({{{{{{\boldsymbol{\omega }}}}}}}_{{{{{{\bf{2}}}}}}}{{{{{\boldsymbol{,}}}}}}{{{{{{\bf{k}}}}}}}_{{{{{{\bf{2}}}}}}}\right){{{{{\boldsymbol{=}}}}}}{{{{{{\boldsymbol{\lambda }}}}}}}_{{{{{{\boldsymbol{1}}}}}}}{{{{{{\boldsymbol{\lambda }}}}}}}_{{{{{{\boldsymbol{2}}}}}}}{{{{{{\boldsymbol{\lambda }}}}}}}_{{{{{{\boldsymbol{3}}}}}}}{{\cong }}{{{{{\boldsymbol{0}}}}}}$, indicating that the SHs should also fall near or even exactly in the inherent modes.

The dispersion relation for plasma waves in a cold plasma can be given by

6 $${\left(\frac{{ck}}{\omega }\right)}^{2}=\frac{B\pm \sqrt{{B}^{2}-4{AC}}}{2A}$$

where

7 $$A=S{\sin }^{2}\theta+P{\cos }^{2}\theta$$

8 $$B={SP}\left(1+{\cos }^{2}\theta \right)+{RL}{\sin }^{2}\theta$$

9 $$C={PRL}$$

with *R*, *L*, *S*, *P* denoting Stix parameters^31^, and $\theta$ denoting the wave normal angle. For whistler-mode chorus waves in the Earth’s inner magnetosphere (${{{{{{\mathbf{\Omega }}}}}}}_{{{{{{\boldsymbol{i}}}}}}}\;{{\ll }}\;{{{{{\boldsymbol{\omega }}}}}}{{{{{\boldsymbol{=}}}}}}{{{{{\boldsymbol{2}}}}}}{{{{{\boldsymbol{\pi }}}}}}{{{{{\boldsymbol{F}}}}}}{{\ll }}{{{{{{\boldsymbol{\omega }}}}}}}_{{{{{{\boldsymbol{pe}}}}}}}$, where ${{{{{{\mathbf{\Omega }}}}}}}_{{{{{{\boldsymbol{i}}}}}}}$ and ${{{{{{\boldsymbol{\omega }}}}}}}_{{{{{{\boldsymbol{pe}}}}}}}$ are the ion cyclotron frequency and electron plasma frequency, and *F* is the wave frequency), we have

10 $$P\approx -\frac{{\omega }_{{pe}}^{2}}{{\omega }^{2}}$$

11 $$R\approx -\frac{{\omega }_{{pe}}^{2}}{\left(\omega+{\Omega }_{e}\right)\omega }$$

12 $$L\approx -\frac{{\omega }_{{pe}}^{2}}{\left(\omega -{\Omega }_{e}\right)\omega }$$

13 $$S\approx -\frac{{\omega }_{{pe}}^{2}}{{\omega }^{2}-{\Omega }_{e}^{2}}$$

14 $$C={PRL}\approx {PSP}$$

15 $$B\approx 2{SP}$$

16 $$A\approx -\frac{{\omega }_{{pe}}^{2}}{{\omega }^{2}\left({\omega }^{2}-{\Omega }_{e}^{2}\right)}\left({\omega }^{2}-{\Omega }_{e}^{2}{{ {\cos }}}^{2}\theta \right)$$

Consequently, the approximated linear dispersion relation for whistler waves can be expressed as

17 $${\left(\frac{{{{{{\boldsymbol{ck}}}}}}}{{{{{{\boldsymbol{\omega }}}}}}}\right)}^{2}=\frac{{{{{{{\boldsymbol{\omega }}}}}}}_{{{{{{\boldsymbol{pe}}}}}}}^{2}}{{{{{{\boldsymbol{\omega }}}}}}(|{{{{{{\boldsymbol{\Omega }}}}}}}_{{{{{{\boldsymbol{e}}}}}}}|{{{{{\bf{c}}}}}}{{{{{\bf{o}}}}}}{{{{{\bf{s}}}}}}{{{{{\boldsymbol{\theta }}}}}}-{{{{{\boldsymbol{\omega }}}}}})}$$

or,

18 $$\frac{F}{{F}_{{ce}}}=\frac{{{\cos }}\theta }{1+{\left({\omega }_{{pe}}/{kc}\right)}^{2}}$$

When both lower-band chorus waves and their SH satisfy the dispersion relation^35,37^,

that is,

19 $$\frac{{\omega }_{1}}{\left|{\Omega }_{e}\right|}=\frac{{{\cos }}\theta }{1+{\left({\omega }_{{pe}}/{k}_{1}c\right)}^{2}}$$

20 $$\frac{{\omega }_{2}}{\left|{\Omega }_{e}\right|}=\frac{{{\cos }}\theta }{1+{\left({\omega }_{{pe}}/{k}_{2}c\right)}^{2}}$$

Note that the necessary conditions for SH generations, as demonstrated in Eq. 4, should be satisfied to result in a nonlinear phase coherence between the fundamental and SHs^17^. Therefore, we can obtain the preferred condition of lower-band chorus to generate SHs

21 $$\frac{F}{{F}_{{ce}}}=\frac{1}{3}{{\cos }}\theta$$

The validation of this preferred condition can be seen in the statistical survey shown in Fig. 1c and the theoretical results shown in Supplementary Fig. 7, both of which confirm that SH can obtain significant energy from the fundamental emission when the preferred condition is satisfied.

### Fokker–Planck simulation

To estimate the evolution of particle phase space density distributions (*f*), it is often to implement the Fokker–Planck simulation, in which the following Fokker–Planck diffusion equation is numerically solved^23–25^

22 $$\frac{\partial f}{\partial t}=	 \frac{1}{G}\frac{\partial }{\partial {\alpha }_{{eq}}}G\left(\left\langle {D}_{{\alpha }_{{eq}}{\alpha }_{{eq}}}\right\rangle \frac{\partial f}{\partial {\alpha }_{{eq}}}+p\left\langle {D}_{{\alpha }_{{eq}}p}\right\rangle \frac{\partial f}{\partial p}\right)\\ 	+\frac{1}{G}\frac{\partial }{\partial p}G\left(p\left\langle {D}_{p{\alpha }_{{eq}}}\right\rangle \frac{\partial f}{\partial {\alpha }_{{eq}}}+\left\langle {D}_{{pp}}\right\rangle \frac{\partial f}{\partial p}\right)-\frac{f}{ \tau }$$

where $\langle {D}_{{\alpha }_{{eq}}{\alpha }_{{eq}}}\rangle$, and $\langle {D}_{{pp}}\rangle$ are the bounce-averaged pitch-angle and momentum diffusion rates, respectively, while $\langle {D}_{{\alpha }_{{eq}}p}\rangle=\langle {D}_{p{\alpha }_{{eq}}}\rangle$ are the mixed terms^19–21^, ${\alpha }_{{eq}}$ is the equatorial pitch angle, *p* is the particle momentum, $G={p}^{2}{{\sin }}{\alpha }_{{eq}}{{\cos }}{\alpha }_{{eq}}(1.30-0.56{{\sin }}{\alpha }_{{eq}})$, and $\tau$ is one quarter of the bounce period for particles  inside the loss cone but it is set to be infinity for particles outside the loss cone.

## Supplementary information

Supplementary Information

## Data Availability

The Van Allen Probes data are publicly available from the Van Allen Probes science center website (http://vanallenprobes.jhuapl.edu/, http://emfisis.physics.uiowa.edu/, https://spdf.gsfc.nasa.gov/pub/data/rbsp/rbspa/l3/ect/). NOAA 18 data that support the findings of this study are publicly available at https://spdf.gsfc.nasa.gov/. The current work uses MATLAB coastline data to generate the global image. The datasets generated during and/or analyzed in the current study are available from the corresponding author upon request.

## Code availability

The computer code used to implement the Fokker–Planck simulation including chorus wave-drive diffusions is available upon request to the corresponding authors.
