# Supplementary material for: Diffuse auroral precipitation driven by lower-band chorus second harmonics
Source: Nat Commun. 2023 Jan 27;14:438. doi: 10.1038/s41467-023-36095-x (PMC9883244; doi:10.1038/s41467-023-36095-x)
Supplement: Supplementary file 1 — Supplementary Information [file 41467_2023_36095_MOESM1_ESM.pdf]

Supplementary Information

Diffuse auroral precipitation driven by lower-band chorus second harmonics

Xiongdong Yu[1], Zhigang Yuan[1*], Jiang Yu[2], Dedong Wang[3], Dan Deng[1], and H. O. Funsten[4]

[1]School of Electronic Information, Wuhan University, Wuhan, Hubei, China

[2]Planetary Environmental and Astrobiological Research Laboratory (PEARL), School of Atmospheric Sciences, Sun Yat-sen University, Zhuhai, China

[3]GFZ German Research Center for Geosciences, Potsdam, Germany

[4] ISR Division, Los Alamos National Laboratory, Los Alamos, NM, USA

* Correspondence and requests for materials should be addressed to Zhigang Yuan (y_zgang@vip.163.com)

Figures

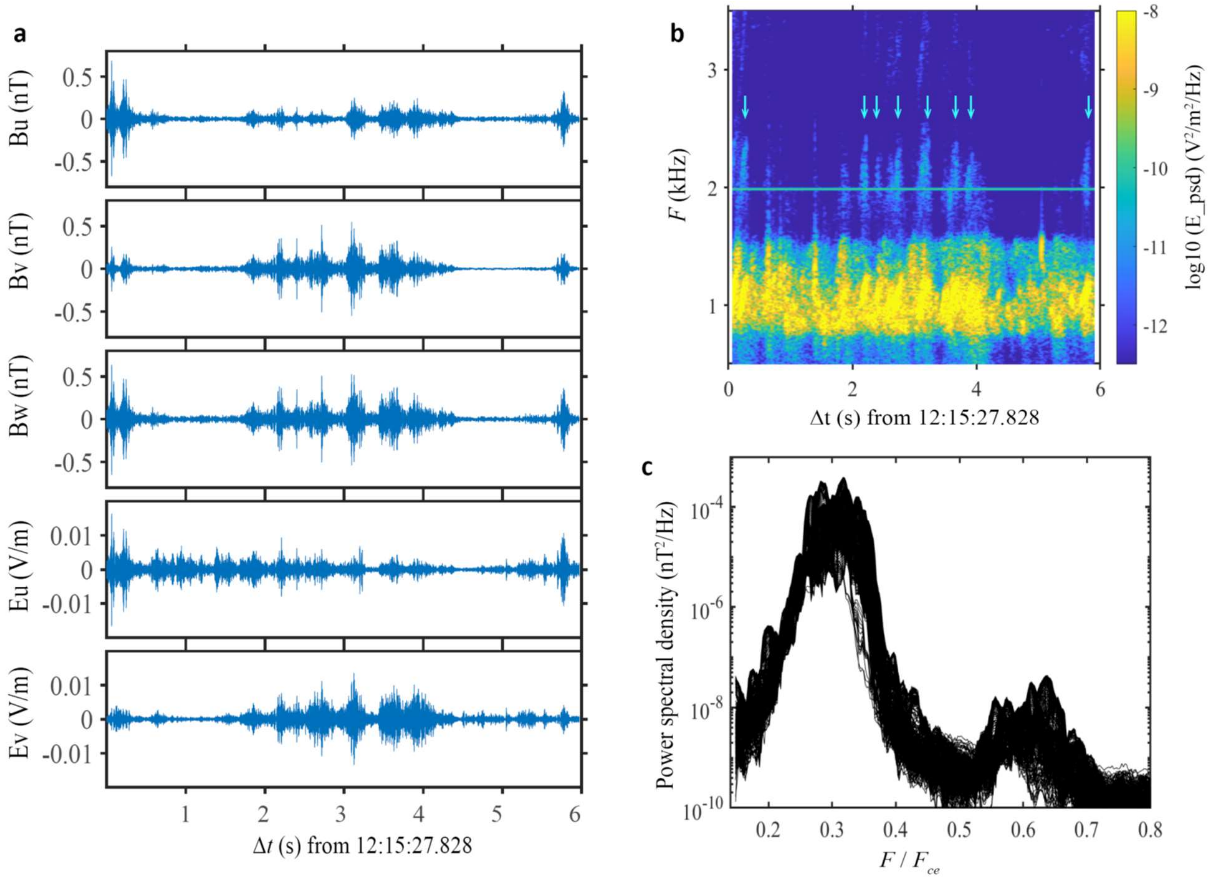

Supplementary Figure 1. Samples of magnetic fields and electric fields (a), the power spectral density of the electric fields (b) and magnetic fields (c). In panel (b), light blue arrows mark the second harmonics of lower-band chorus waves. The observed magnetic field components (peak-to-peak) are about 300–700 pT, and the electric field components (peak-to-peak) are about 10 mV/m. The latter is only about 1/40 of the range of EFW instrument, but second harmonics in the electric field spectra are found to occur in a same way as in the magnetic field one.

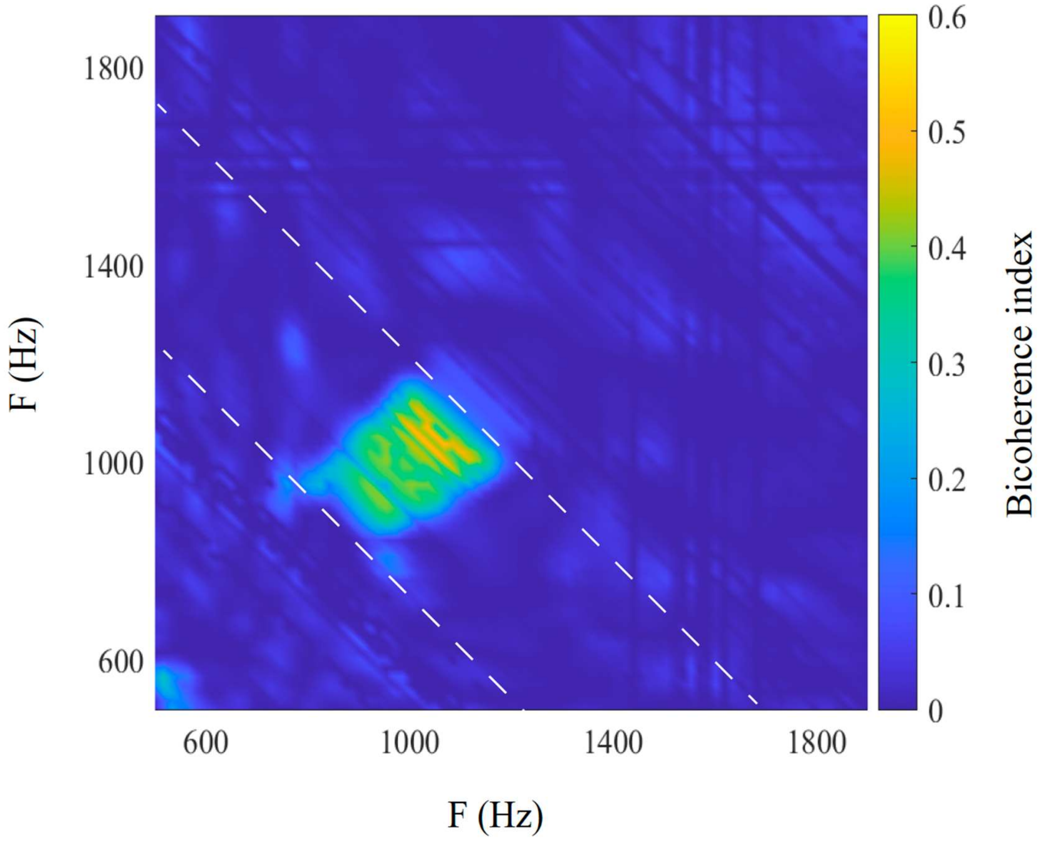

Supplementary Figure 2. Bicoherence index calculated from the detected magnetic and electric fields near 12:15:17 UT. High bicoherence index is found near 1000 Hz in both abscissa and ordinate, implying strong wave-wave coupling for lower-band chorus and their second harmonics.

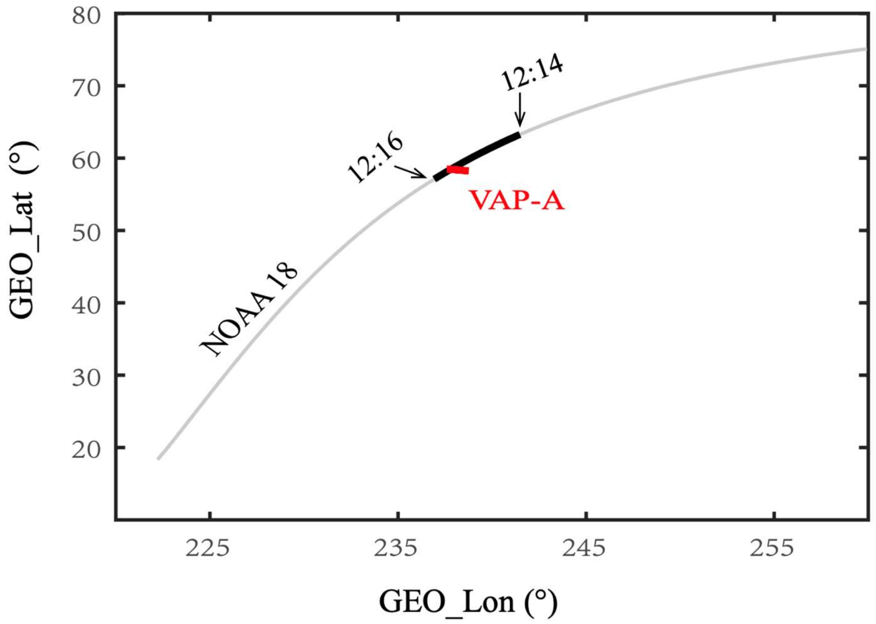

Supplementary Figure 3. Footprints of Van Allen Probe A and NOAA 18 between 1210 UT and 1230 UT. National Oceanic and Atmospheric Administration Polar Orbiting Environmental Satellite 18 (NOAA 18) has captured diffuse auroral electron precipitation in the regions (bold black curve) conjugately with Van Allen Probe A (VAP-A), in which lower-band chorus and their second harmonics have been found as well as electron pancake distributions (bold red curve).

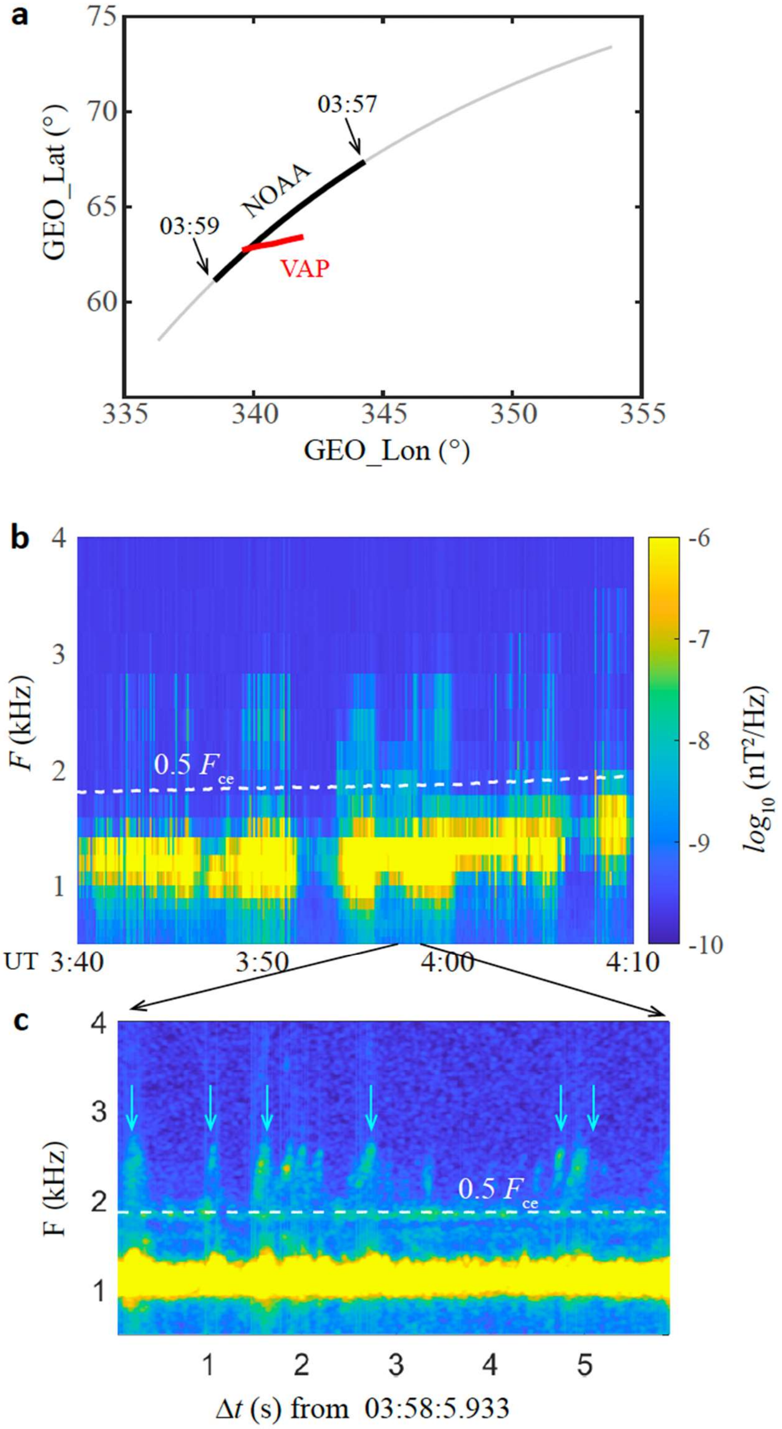

Supplementary Figure 4. Van Allen Probe observations of another simultaneously observational event of in January 20, 2013. (a) Footprints of National Oceanic and Atmospheric Administration Polar Orbiting Environmental Satellite 19 (NOAA 19), and Van Allen Probe A (VAP-A). (b)-(c) chorus wave power spectral densities in survey and burst modes measured by the Van Allen Probe A. In panel (c), light blue arrows mark the observed second harmonics.

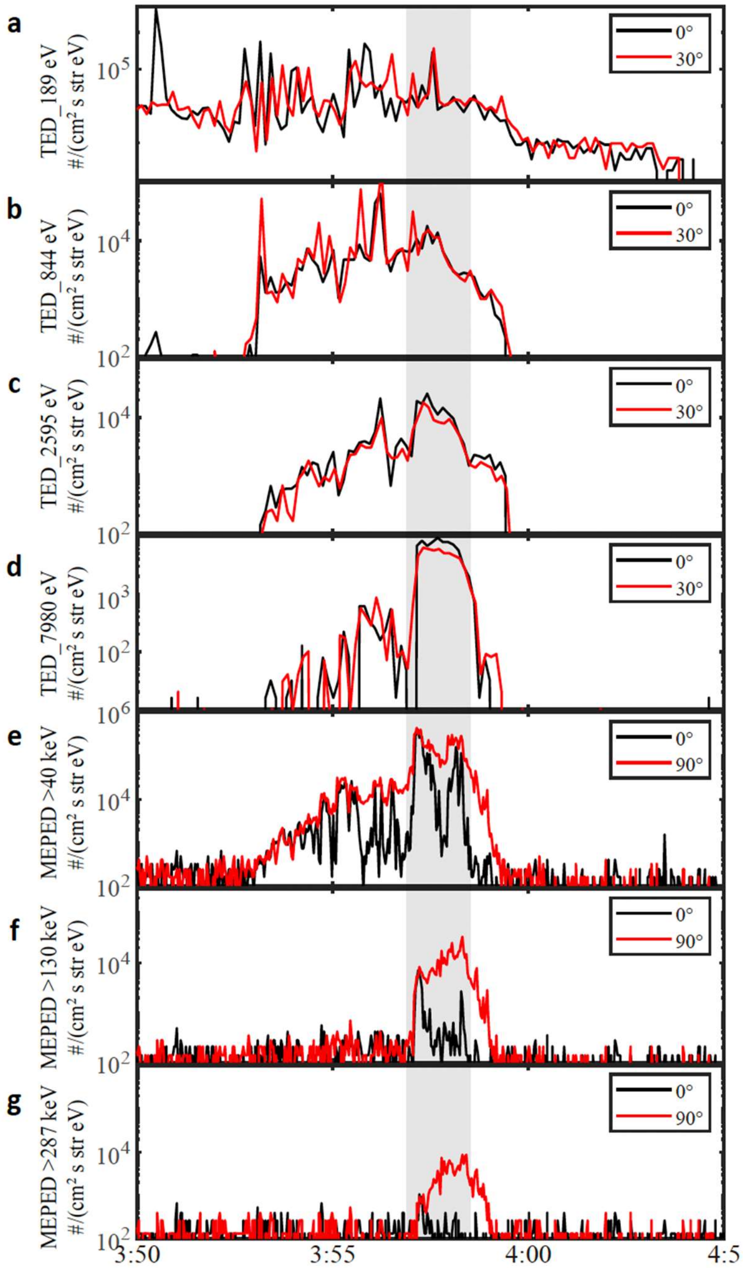

Supplementary Figure 5. NOAA **observations of another simultaneously observational event of in January 20, 2013.** (a)-(d) Precipitating electron fluxes in different energy channels (189 eV, 844 eV, 2.595 keV, and 7.980 keV, respectively) measured by the Total Energy Detector (TED) onboard National Oceanic and Atmospheric Administration Polar Orbiting Environmental Satellite 19 (NOAA 19) in the ionospheric altitude. (e) -(g) Precipitating electron fluxes in different energy channels (>40 keV, >130 keV, and >287 keV, respectively) measured by the Medium Energy Proton and Electron Detector (MEPED) onboard NOAA 19. The grey rectangle in indicates the electron diffuse auroral precipitation observed by NOAA 19 in the ionospheric position conjugated with Van Allen Probe A which has detected chorus waves and their second harmonics near the magnetospheric equator.

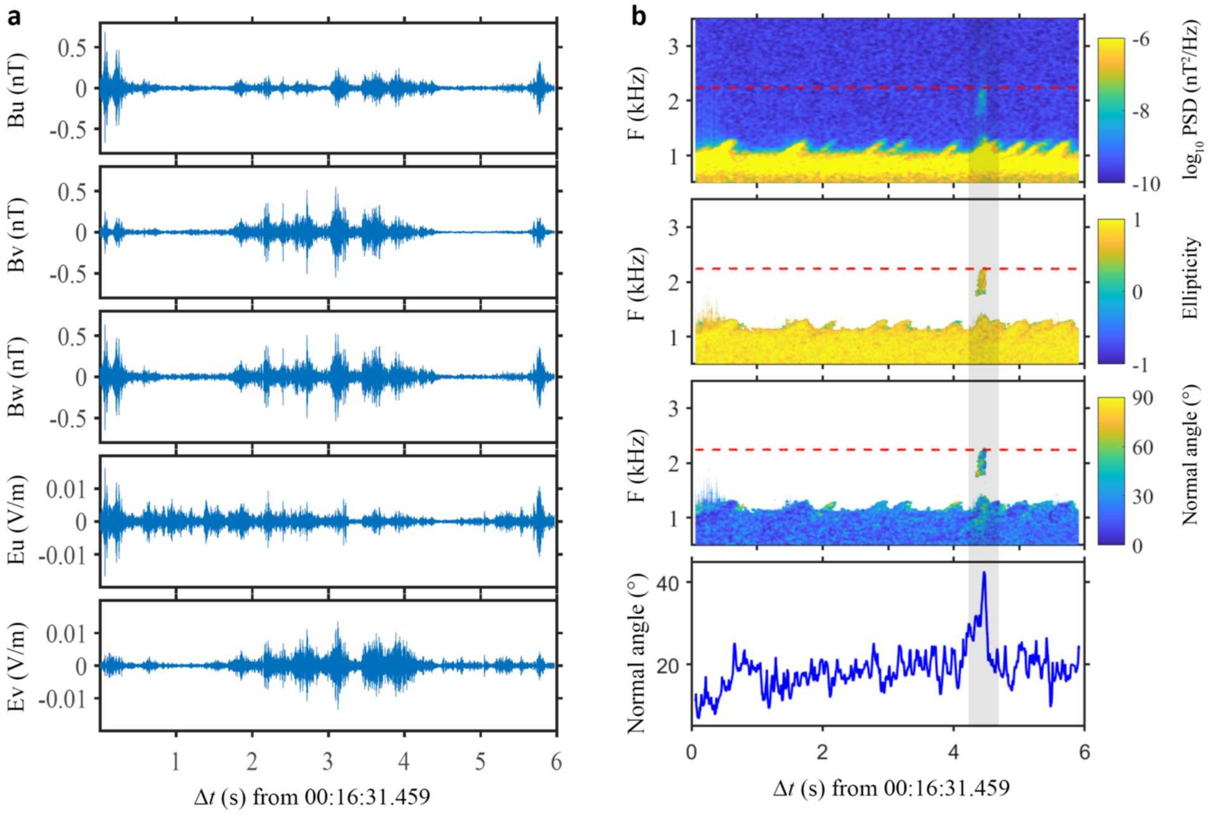

Supplementary Figure 6. Another second harmonic event observed by the Van Allen Probe A in February 24, 2014. (a) Samples of tri-axis magnetic fields (Bu, Bv, Bw) and electric fields in the spin plane (Eu, Ev) measured in the burst mode. (b) Power spectral densities of the measured magnetic fields, and their corresponding ellipticity, normal angles, and power-weighted normal angles. Chorus element accompanied with second harmonics are marked by the grey rectangles.

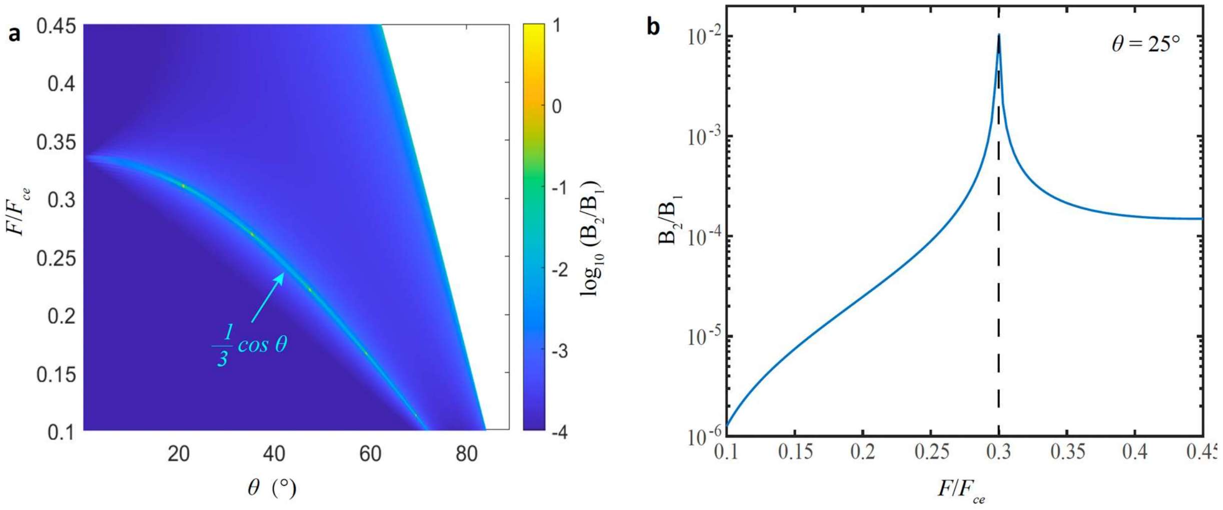

Supplementary Figure 7. Theoretical amplitude ratio of the second harmonic to the fundamental wave. (a) Amplitude ratio (B_2/B_1) as a function of normalized frequency and normal angles, using the plasma environment observed in Figure 1b and the observed amplitude of the fundamental wave (B_1 = 0.35 nT). (b) A ratio profile at a normal angle of 25° (θ = 25°). The theoretical ratio becomes significantly large near the preferred condition (F/F_{ce} = $\cos\theta/3$), of which this ratio owns a peak value of 1.04% when the normalized frequency of the fundamental harmonic is 0.3. It is suggested that the observed second harmonics are excited by the fundamental through nonlinear wave processes.

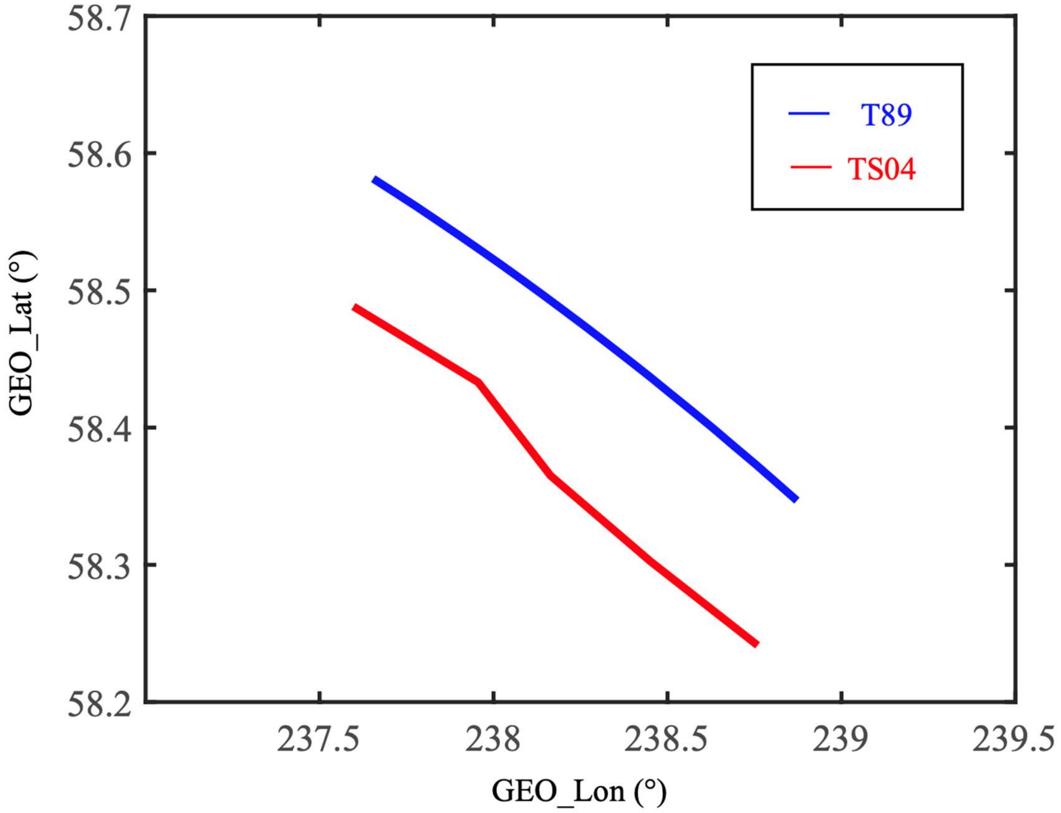

Supplementary Figure 8. Footprints of the Van Allen Probe A obtained from Tsyganenko magnetic models T89 (blue), and TS04D (red) in geophysical latitudes (GEO_Lat) and longitudes (GEO_Lon).
